# Cocreation, Implementation, and Evaluation of Two Digital Innovations to Improve Home Adaptation for Older Adults: Protocol for an Action Research Pilot Study

**DOI:** 10.2196/84680

**Published:** 2026-09-03

**Authors:** Lucien Coulibaly, Maude Laberge, Manon Guay, Noémie Séguin-Tremblay, Paule Langlois, Isabel Paradis, Jean Philippe Nadeau, Mireille Jobidon, René Chamard, Karine Latulippe

**Affiliations:** 1 Department of Social and Preventive Medicine, Faculty of Medicine, Université Laval, 2325 Rue de l'Université, Quebec, QC, Canada; 2 Université Laval Research Centre, Population Health and Best Health Practices Axis, CHU de Québec, Quebec, QC, Canada; 3 Vitam, Centre for Research in Sustainable Health, Université Laval, Quebec, QC, Canada; 4 Department of Occupational Therapy, Université du Québec à Trois-Rivières, Trois-Rivières, QC, Canada; 5 Research Centre on Aging, Université de Sherbrooke, Sherbrooke, QC, Canada; 6 Department of Education, Educational Technology Program, Université TÉLUQ, Quebec, QC, Canada; 7 Department of Education, Educational Technology Program, Université TÉLUQ, 455 Rue du Parvis, Quebec, QC, G1K 9H6, Canada, 1 418-657-2262 ext 5162, 1 418-435-8541; 8 Bas-Saint-Laurent Integrated Health and Social Services Centre, Saint-Épiphane, QC, Canada; 9 Bas-Saint-Laurent Integrated Health and Social Services Centre, Quebec, QC, Canada; 10 Quebec Housing Corporation, Quebec, QC, Canada; 11 Citizen Partner with Experience as a Home Adaptation Program (HAP) User, Quebec, QC, Canada; 12 Quebec Centre for Research and Innovation in Educational Technology (i-TEQ), Quebec, QC, Canada

**Keywords:** older adults, home adaptation, innovation, Hygiene 2.0, MapIt, action research, cocreation

## Abstract

**Background:**

Aging in place is a priority for many older adults but often requires home adaptations when temporary or permanent physical disabilities arise. It reduces the risk of falls, improves health-related quality of life, and enhances social participation. Two new digital innovations aim to support home adaptation: Hygiene 2.0, which facilitates the adaptation of baths and showers, and MapIt, which provides 3D mapping of the environment.

**Objective:**

This action study aims to cocreate and evaluate the implementation of the Hygiene 2.0 and MapIt innovations and to measure their impact on supporting older adults who experience difficulties with personal hygiene or are in the process of adapting their homes.

**Methods:**

This study uses a multiple case study approach with a mixed convergent design, guided by the i-PARIHS (integrated-Promoting Action on Research Implementation in Health Services) framework. We will conduct semistructured interviews with 66 participants, including 8 older adults-informal caregiver dyads (or individual older adults when no informal caregiver is available) for MapIt only, 20 dyads for Hygiene 2.0 or the combined use of Hygiene 2.0 and MapIt, and 10 assistive technology advisors across all innovation scenarios. A cost analysis will be conducted from both the Quebec public system and patient perspectives. Qualitative data and mixed data integration will be analyzed using analytical questioning.

**Results:**

This study was conducted within the framework of, and with financial support from, the *Bien vieillir chez soi au Bas-Saint-Laurent* action research program. Recruitment began in May 2024. We completed data collection in February 2026 and began data cleaning and analysis in March 2026. As of September 2025, we enrolled 1 dyad of older adults and informal caregivers using MapIt, 5 dyads using Hygiene 2.0 or the combination of Hygiene 2.0 and MapIt, and 10 assistive technology advisors. The manuscript describing the results of the study is expected to be published at the beginning of 2027. May.

**Conclusions:**

This study will provide valuable insights into how innovative digital tools can facilitate home adaptations that promote aging in place. By assessing their implementation, usability, and cost-effectiveness, the findings are expected to inform best practices, guide decision-making for health and social care stakeholders, and ultimately contribute to improving the quality of life and autonomy of older adults.

## Introduction

### Context

The home is more than a physical space; it is a crucial determinant of health and a place of belonging [1]. When older adults experience functional decline, most of them continue to live in traditional homes (eg, houses or apartments), which often reflect their preferred choice of residence [2]. However, challenges may arise in performing essential daily activities, such as personal hygiene [3], especially when the home environment has not been adapted to meet their evolving needs. An adapted living environment significantly contributes to enabling older adults to age in place safely. It helps reduce falls, enhances health-related quality of life, and increases social participation [4-6].

A quasi-experimental (before-and-after) study by Petersson et al [7] demonstrated a statistically significant improvement in self-rated ability in everyday life in the intervention group (who received home adaptations) compared to the control group (on a waiting list). Participants who benefited from the home improvement program reported fewer difficulties and increased safety when performing personal hygiene tasks in the bathroom. Similarly, a prospective quasi-experimental (before-and-after) study showed that home modifications led to significant and sustained improvement in the participants’ perception of their ability to carry out daily activities at home over a 2-year period. The authors concluded that home adaptations can be beneficial for older adults wishing to age in place [2].

The search for environmental design solutions is often initiated by older adults or their families when bathroom-related issues arise. In such cases, adapting the bathroom with assistive devices, such as bath seats or grab bars, is a commonly recommended strategy to promote safety and autonomy [8,9]. Moreover, home modifications are frequently recommended by occupational therapists to reduce environmental barriers and improve the functional performance of individuals with limitations [10].

### Description of Current Practice

In Quebec, various options are available for older adults who wish to adapt their home environment. They may obtain recommendations from companies that provide assistive devices, such as pharmacies or specialized suppliers. It is also possible to access the services of occupational therapists within the public health and social services system or to consult occupational therapists in private practice. When home modifications are more extensive and the older adult has permanent disabilities, an application can be submitted to the *Home Adaptation Program* (HAP), a government initiative that partially or fully covers the costs of necessary adaptations (eg, zero-threshold shower and access ramp).

Despite the availability of these resources, older adults and their families often face significant challenges, such as long waiting times, complex administrative processes, and limited accessibility to professional support. These barriers highlight the need to explore alternative and complementary approaches that can provide timely, accessible, and practical guidance.

In this context, digital technologies are emerging as promising tools to help circumvent or mitigate several of these obstacles. By providing autonomous assessment tools and facilitating the collection and sharing of essential information, digital innovations can support older adults and their caregivers in decision-making while reducing reliance on lengthy and centralized health care processes. Two digital innovations, Hygiene 2.0 [11] and MapIt [12], illustrate how technological approaches can directly address these challenges and support the adaptation of older adults’ homes.

### Description of Innovations

The Hygiene 2.0 application [11] aims to allow for the rapid identification of appropriate assistive devices for bathroom adaptations (eg, shower chair and grab bars) and target persons needing occupational therapist consultations. It thus helps accelerate access to practical solutions for older adults, reducing the pressure on professional services. The MapIt mobile app [12] allows users to generate a photorealistic 3D model of a room in just a few minutes, facilitating remote assessment, information sharing with various stakeholders, and the preparation of more effective interventions.

### Research Objectives and Questions

The objective of this action study is to cocreate and evaluate the implementation within the ecosystem of older adults of the Hygiene 2.0 and MapIt innovations, as well as to assess their impact on supporting older adults who experience difficulties with personal hygiene or who are in the process of adapting their home environment. Specifically, the objectives of the study are as follows:

1. Objective 1: To describe the cocreation process for implementing Hygiene 2.0 and MapIt and identify facilitators and barriers to implementation.
  1.1: How does the cocreation process unfold among the various stakeholders involved in the implementation of Hygiene 2.0 and MapIt?
  1.2: What are the main facilitators and barriers encountered during the implementation of Hygiene 2.0 and MapIt?
2. Objective 2: To examine the perceived experiences of various user groups and stakeholders involved with these innovations.
  2.1. How do different user groups perceive their experience using Hygiene 2.0 and MapIt?
  2.2. What are the factors that influence their experiences?
  2.3. Do perceptions and experiences differ across stakeholder groups?
3. Objective 3: To explore preliminary outcome patterns related to occupational outcomes, quality of life, and implementation-related costs associated with the use of Hygiene 2.0 and MapIt.
  3.1. What preliminary patterns in users’ occupational outcomes are observed following the implementation of Hygiene 2.0 and MapIt across different contexts?
  3.2. What preliminary changes in quality of life are observed among individuals following exposure to the innovations?
  3.3. What are the direct and indirect costs associated with the implementation of Hygiene 2.0 and MapIt, and how do these costs inform an initial appraisal of potential efficiency and outcome-related benefits?

## Methods

### Theoretical Framework: I-PARIHS Model

The i-PARIHS (integrated-Promoting Action on Research Implementation in Health Services) framework [13] will guide this study by identifying and defining the characteristics of innovations, as well as informing the facilitation strategies needed to support their use within the ecosystem of older adults engaged in home adaptation processes. The model proposes that the successful implementation of an innovation results from the interaction between 4 key elements: innovation (what is being implemented, eg, a new practice), recipients (individuals, teams, or organizations expected to adopt the innovation), context (the internal and external environment in which the innovation is implemented), and facilitation (the active process of enabling and supporting implementation). In essence, the i-PARIHS model conceptualizes successful knowledge implementation as the result of effectively facilitating innovation, in collaboration with recipients, within the context in which the implementation occurs. This approach emphasizes the dynamic interplay between the innovation, the recipients, the context, and the facilitation process.

### Study Design

The pilot study is grounded in participatory action research (PAR), an approach that values collaboration between researchers and practitioners. It emphasizes colearning, mutual benefit, and long-term engagement. PAR integrates theory, participation, and practice within the research process [14]. More than simply involving the community, it aims to connect participation, shared decision-making, local knowledge, and existing practices in a meaningful process of change. This approach is particularly well suited to this study, as it enables the inclusion of diverse stakeholders (older adults, informal caregivers, researchers, and organizational representatives) in cocreating the implementation process, while fostering collaboration firmly anchored in real-world community contexts. The cocreation process for implementing the MapIt and Hygiene 2.0 innovations involves exploring the relevance and appropriateness of their use with various stakeholders, including older adults. In this study, implementation fidelity is defined at the level of the digital tools’ core functional components rather than uniform patterns of use. MapIt and Hygiene 2.0 are designed as decision-support technologies whose technical execution is standardized, while their integration into practice may vary according to context, user roles, and situational needs. Such variability is considered an intended feature of implementation, consistent with the study’s design emphasizing practitioner and user agency.

To operationalize this participatory approach and capture the complexity of real-world contexts, the study employs a multiple case study design with a convergent mixed methods strategy [15,16]. This design integrates the strengths of both quantitative and qualitative methods [17]. Each “case” will represent either an older adult undergoing a home adaptation process or an assistive technology advisor (eg, an occupational therapist) using one or both digital innovations.

### Study Population

Participants will include older adults and their informal caregivers, assistive technology advisors (eg, occupational therapists), and representatives from Regional County Municipalities responsible for implementing the HAP.

### Eligibility Criteria

Participants will include older adults, informal caregivers, and assistive technology advisors. Older adults’ inclusion criteria are as follows: (1) aged 55 years or older, (2) residing in the Bas-Saint-Laurent (BSL) region, (3) deemed clinically capable of providing informed consent, and (4) expressing having difficulty or fear related to bathing or showering. Informal caregivers refer to a person who provides unpaid care and assistance to someone with a chronic illness, disability, or other long-lasting health or personal care needs, typically a family member or close friend. No specific number of support hours is required. An assistive technology advisor is a person who provides guidance to older adults in adapting their home or bathroom (eg, occupational therapist, personal support worker, and assistive device consultant).

### Sample

A nonprobability convenience sampling method will be used [18]. The target sample includes 66 participants: 8 dyads of older adults and informal caregivers using MapIt, 20 dyads using Hygiene 2.0 or the combination of Hygiene 2.0 and MapIt, and 10 assistive technology advisors using at least one of both digital tools. This sample size is justified by the recruitment challenges and the exploratory nature of this pilot study [11], which primarily aims to assess feasibility, refine methodological procedures, and identify necessary adjustments before conducting a larger-scale study.

### Recruitment Process

Except for the case where older adults use Hygiene 2.0 independently, participant recruitment will be carried out through intermediaries. For assistive technology advisors and occupational therapists, recruitment will occur via their employers or affiliated organizations. Assistive technology advisors may refer eligible older adults and informal caregivers whom they have supported. If older adults verbally agree, the advisors will forward their contact information to the research team so that the team members may contact them to provide additional information about the project. For older adults and informal caregivers using Hygiene 2.0 independently, recruitment will involve posters (see Multimedia Appendix 1) displayed in community spaces, such as pharmacies and post offices. The project will also be promoted through local media (eg, municipal newspapers) and community groups serving older adults.

### Ethical Considerations

This pilot study was approved by the research ethics board of the BSL Integrated Health and Social Services Center (project 2024‐459), as well as the Université TÉLUQ (project 2024‐80) and Université Laval (project 2024‐345) research ethics boards. In all cases, a member of the research team will introduce the project, answer questions, and obtain written informed consent.

For Hygiene 2.0, the data collected will be limited to user responses, associated with an anonymized digital identifier, and no directly identifiable personal data (eg, name, address, image, or IP address) will be collected. All data will be stored on a secure institutional server in accordance with ethical standards and governance policies. For MapIt, the digitization of home environments is performed only after obtaining informed consent, and the digital representations remain stored locally on the device without automatic transfer to external servers. User training will emphasize confidentiality principles, including limited data retention periods, and the systematic deletion of data once its intended use has been fulfilled. When sharing with occupational therapists is required, files will be transmitted via a secure server through an institutional Microsoft Teams environment that complies with health data security standards.

### Conduct of the Study and Method of Data Collection in Different Contexts

**Figure 1.**
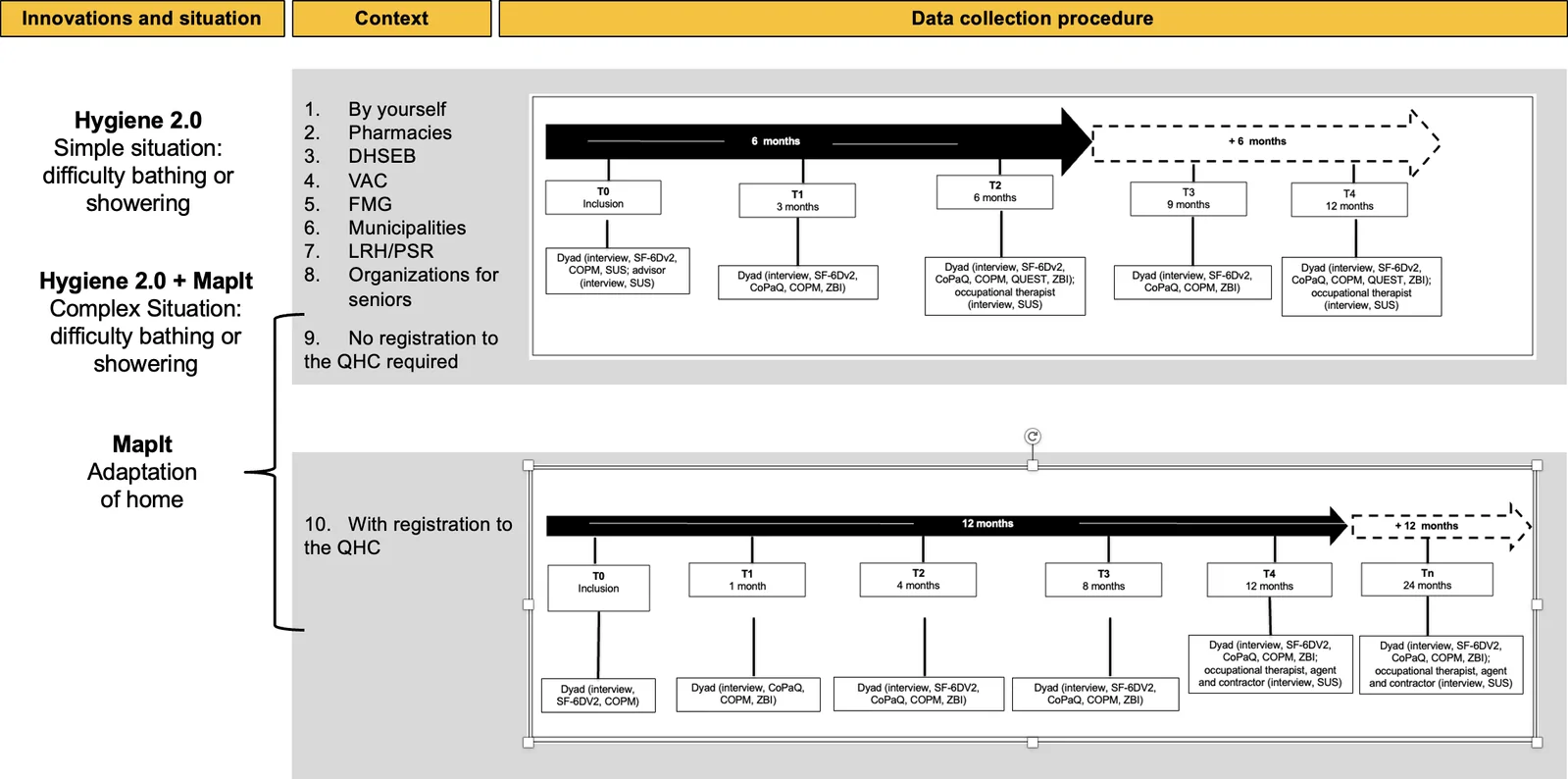
Conduct of the study. CoPaQ: Costs for Patients Questionnaire; COPM: Canadian Occupational Performance Measure; DHSEB: domestic help social economy business; FMG: family medicine group; LRH: low-rental housing; PSR: private older adults’ residence; QHC: Quebec Housing Corporation; QUEST: Quebec User Evaluation of Satisfaction with Assistive Technology; SF-6DV2: Short Form-6 Dimensions, version 2; SUS: System Usability Scale; VAC: volunteer action center; ZBI: Zarit Burden Interview.

The project is led by a steering committee composed of 11 partners from the health and social services network, home adaptation services, research, and a citizen partner. Different implementation scenarios will be explored: the Hygiene 2.0 and MapIt innovations, individually or in combination, will be implemented across 3 distinct scenarios encompassing 10 contexts (Figure 1).

The term “contexts” refers to the variety of service points where older adults may access these tools, either independently or with support. These include settings where they are introduced to Hygiene 2.0 or receive assistance with home adaptation planning through MapIt. Three primary situations have been identified, each representing a distinct configuration of needs and interventions and involving the use of Hygiene 2.0, MapIt, or a combination of both: (1) simple situation—difficulty with personal hygiene: Hygiene 2.0 is used to support older adults who experience challenges with bathing or showering, without the need for broader home adaptation; (2) complex situation—difficulty with personal hygiene and home adaptation needs: a combined use of Hygiene 2.0 and MapIt is applied for older adults facing both hygiene-related difficulties and requiring home modifications; and (3) home adaptation only: MapIt is used independently to support the adaptation of the home environment for older adults who do not currently face hygiene challenges but are undergoing home modifications.

### Implementation and Evaluation

We will document which stakeholders adopt the tools, how their use is operationalized, how frequently they are used, and the benefits perceived by users. Additionally, we will track the reasons for nonuse or refusal to adopt the innovations, as well as the facilitators and barriers to their implementation. Two barriers have already been identified by the steering committee, which allowed for the immediate development of solutions: (1) a training module for the use of MapIt, available free of charge and online through the website of the Quebec Order of Occupational Therapists and (2) the loan of equipment to ensure access to the applications, including electronic tablets (iPad Pro) and smartphones (iPhone 16). Data sources will include research team logbooks, accountability reports, and records of participation in training sessions and presentations of the innovations (Multimedia Appendix 2).

### Evaluation of Implementation Impact

To evaluate the impact of implementation, the data will be collected from the inclusion point (T0) of older adults in the study, using the mode of communication they prefer (online, by telephone, or in person).

For the Hygiene 2.0 innovation, applied in simpler situations involving challenges with bathing or showering (contexts 1-8 in Figure 1), the study period will span 6 months. Data will be collected at baseline, 3 months, and 6 months (T0, T1, and T2), which should be sufficient to capture potential clinical improvements. In addition, clinical-administrative databases from local community service centers will be consulted to obtain information on older adults who have received occupational therapy recommendations for bathroom or home adaptations.

In cases involving the combined use of both innovations (MapIt+Hygiene 2.0), the study period will range from 6 to 12 months. This applies to individuals experiencing more complex challenges with bathing or showering that require occupational therapy assessments (contexts 1-8), as well as those involved in home adaptation without HAP (context 9). Data will be collected every 3 months up to 6 months (T0, T1, and T2), with the possibility of extending to 12 months (T3 and T4), if there are delays in accessing occupational therapy services.

For home adaptation cases involving the HAP (context 10), the study period will extend to 24 months, reflecting the typically longer intervention timelines. In these cases, the data will be collected at 4-month intervals beginning at T0.

### Interviews

Semistructured interviews (Multimedia Appendix 3) will be conducted with dyads (older adults and informal caregivers, where applicable) as well as with assistive technology advisors. These interviews aim to explore participants’ experiences with the innovations (MapIt or Hygiene 2.0) and the home adaptation process. Each interview with an older adult and informal caregiver is expected to last up to 60 to 90 minutes, whereas interviews with assistive technology advisors will last up to approximately 45 minutes. When the same assistive technology advisor works with multiple older adults, a brief 5- to 10-minute follow-up interview will be conducted to capture additional insights.

### Variables and Measurement Tools

We focused on a cost analysis [19] only and a longitudinal examination of quality-adjusted life years (QALYs) within participants, rather than on incremental cost-effectiveness or cost-utility analyses.

### Cost

Costs will be categorized into 2 main variables: direct costs and indirect costs (Table 1). Direct costs include the remuneration of occupational therapists, authorized agents (mandataries), and expenses related to the implementation of the innovations and the HAP. Indirect costs refer to out-of-pocket expenses incurred by older adults and their informal caregivers, as well as any additional expenses related to participation in the HAP (eg, adaptation solutions not covered by the HAP).

**Table 1.** Direct costs and indirect costs.

Cost category and cost type	Components
Direct costs	
Professional remuneration	Occupational therapists’ and authorized agents’ hourly rates (including social benefits)
Implementation costs	Resources related to the deployment of innovations and the HAP^a^
Travel costs	Time and distance for home visits converted into monetary values
Indirect costs	
Out-of-pocket expenses	Expenses incurred by older adults and informal caregivers (collected using the CoPaQ)^b^
Participation-related costs	Additional adaptation solutions not covered by the HAP

a HAP: Home Adaptation Program.

b CoPaQ: Costs for Patients Questionnaire.

### Costs for Patients Questionnaire

Cost data for older adults and informal caregivers will be collected using a validated tool called the Costs for Patients Questionnaire (CoPaQ) [20-22]. The CoPaQ was developed through a systematic review and a Delphi process, ensuring strong content validity. Its psychometric properties have been evaluated in multiple studies. Test-retest reliability analyses report intraclass correlation coefficients (ICCs) that ranged from −0.02 to 0.99 (median 0.62) and κ coefficients that ranged from −0.11 to 1.00 (median 0.86), indicating moderate-to-excellent reliability for most items. The structure and wording of the questions were validated to guarantee clarity and relevance for patients and caregivers. These findings confirm that the CoPaQ is a robust instrument for measuring costs borne by patients and their caregivers in economic evaluations.

### Utility and Quality of Life

Utility, expressed as QALYs, enables the integration of both the quality and quantity of life into a single measure. QALYs will be assessed using the Short Form-6 Dimensions, version 2 (SF-6Dv2), a validated, generic, health-related quality-of-life questionnaire [23]. It includes 6 dimensions: physical functioning, role limitations, social functioning, pain, mental health, and vitality. Test-retest reliability analyses report ICC for SF-6Dv2 utility scores as 0.866, indicating good reliability [24]. Among individual dimensions, the physical functioning dimension shows the highest reliability (Gwet agreement coefficient=0.669). The SF-6Dv2 demonstrates a high responsiveness (standardized response means=0.788). The standardized response means is a distribution-based index that quantifies sensitivity to change by standardizing the mean difference with respect to the variability of change scores. The average minimal clinically important difference value for the SF-6Dv2 is 0.078 [25].

### Occupational Performance and Satisfaction

Occupational performance and satisfaction will be measured using the Canadian Occupational Performance Measure, research version [26]. This semistructured tool is recognized for its content validity, established through expert consensus and patient involvement, and construct validity, demonstrated by correlations with functional measures. The instrument shows strong test-retest reliability (ICC values typically >0.80) and moderate interrater reliability, ensuring consistency across administrations [27]. Responsiveness is considered moderate to high, with a minimal clinically important difference of 2 points [28], supporting its sensitivity to change in clinical interventions. These properties make the Canadian Occupational Performance Measure a robust tool for assessing occupational performance and satisfaction among older adults.

### Satisfaction With Assistive Devices

Satisfaction will be assessed using the Quebec User Evaluation of Satisfaction with Assistive Technology (QUEST), which uses a 5-point Likert scale [29,30]. The QUEST psychometric properties are well established. The tool demonstrates excellent reliability (ICC 0.82-0.91) and consistency between self-administered and interviewer versions (ICC 0.76-0.91). Content validity was ensured through input from both individuals who use assistive devices and international experts.

### Informal Caregiver Burden

Informal caregiver burden will be measured using the French 12-item short version of the Zarit Burden Interview (ZBI) [31-33]. It evaluates the emotional and material burden on the primary informal caregiver, using a 5-point scale ranging from 0 (“never”) to 4 (“almost always”). This tool will be used only when an informal caregiver is involved in supporting the older adult. The ZBI is a valid and reliable instrument for measuring the burden experienced by caregivers. The Cronbach α value for the ZBI items is 0.93, and the intraclass correlation coefficient for the test-retest reliability of the Zarit burden score is 0.89 [34].

### Usability of Innovations

Usability will be assessed with the System Usability Scale (SUS) [35], a widely used tool consisting of 10 items rated on a 5-point scale. The SUS has excellent psychometric properties [36]. Research has consistently shown the SUS to have reliabilities at or just over 0.90, far above the minimum criterion of 0.70. The SUS has also been shown to have acceptable levels of concurrent validity and sensitivity [36]. The French version of the SUS demonstrates high reliability [37]. If a single assistive technology advisor supports multiple older adults, the SUS will be completed only once by that assistive technology advisor.

### Data Sources

Occupational therapists will complete a structured logbook (Multimedia Appendix 4) to document their use of MapIt, including context and observed impacts [38]. An online questionnaire (Multimedia Appendix 5) will be administered to older adults or informal caregivers after completing Hygiene 2.0 or viewing its associated videos.

### Process Documentation

To track implementation changes and decisions, research professionals will maintain a logbook [39], recording key decisions, challenges, facilitators, and successes. Steering committee meeting minutes will be used to document the project’s evolution and provide a shared reference for committee members. These minutes will also capture implementation processes, challenges, solutions, and contextual factors influencing the adoption of the innovations.

### Data Analysis

#### Cost Measurement

The cost analysis will follow the CHEERS (Consolidated Health Economic Evaluation Reporting Standards) framework [40]. This analysis will be conducted from 2 analytical perspectives: that of the Quebec public health system and that of patients. For each innovation and context (Hygiene 2.0, the combination of Hygiene 2.0 and MapIt, and MapIt alone), the team will estimate the associated costs over the study period. This will include estimating implementation costs (eg, acquisition of innovations, service usage, and staff remuneration). Remuneration for occupational therapists and authorized agents will be calculated based on their standard hourly rates. Travel distance and time for home visits will be converted into monetary values. We will present the descriptive statistics of participants’ basic characteristics across the full sample and calculate total and average costs, valued in Canadian dollars for the base year (2025). Because the cost collection period is less than 1 year for Hygiene 2.0 and 6 months for the combined Hygiene 2.0 and MapIt interventions, no discounting will be applied. However, for the MapIt-alone intervention with Quebec Housing Corporation registration, where the data collection period spans more than 1 year, a discount rate will be applied. For this specific context (context 10), we will also analyze secondary cost data from the HAP database over the past 2 years. For each of the innovations and contexts, we will also estimate QALYs accrued during the study period and analyze variations.

#### Qualitative and Mixed Methods Analyses

A qualitative analysis based on analytical questioning [41] will be conducted on interview transcripts (fully transcribed from audio recordings), research professionals’ diaries, and project reports. This analysis will be guided by the i-PARIHS framework. We will use the NVivo software (Lumivero) to assist with the organization and coding of qualitative data. The qualitative and quantitative data will be integrated through an analytical questioning approach embedded within a convergent mixed methods design. A set of analytical questions will be defined a priori, reflecting the study objectives and the underlying conceptual framework. These analytical questions (Multimedia Appendix 6) will guide both the initial qualitative analysis and the subsequent integration of quantitative data, ensuring alignment with the i-PARIHS framework. Rather than being analyzed and interpreted in isolation, qualitative and quantitative datasets will be jointly mobilized to address each analytical question, allowing findings from both strands to be examined in relation to one another within a single, convergent analytical phase. This approach enables the systematic identification of convergence, complementarity, and divergence across data types and supports a fully integrated interpretation of implementation processes and preliminary outcome patterns. To strengthen the interpretation of findings, the triangulation of qualitative and quantitative data will be carried out using a mind map approach. For each key element (innovation, recipients, context, and facilitation) of the i-PARIHS model, the qualitative and quantitative findings will be synthesized and compared to provide a richer understanding of the contributions and limitations of the innovations and their implementation within the ecosystem of older adults in the BSL region. The analysis will be led by the research team and subsequently validated by the steering committee.

#### Statistical Analyses

All analyses will be conducted using Stata (version 17; StataCorp LLC), with the significance level set at 0.05. QALYs will be estimated for each participant using the area-under-the-curve method over the follow-up period. Changes in costs and utility values over time will be examined using a within-subject nonparametric test (Friedman test), given the repeated-measures design, the small sample size, and the nonnormal distribution of the data. If a statistically significant overall effect of time is observed, post hoc pairwise comparisons will be performed to identify the specific measurement time points between which differences occur. All relevant pairwise comparisons between measurement time points (T0-T4) will be performed to identify specific differences over time. To control for the inflation of the type I error rate due to multiple testing, the Bonferroni correction will be applied. Dichotomous and nominal variables will be summarized using frequencies and percentages. Missing data will be assessed for their extent and patterns. When the data are assumed to be missing at random, multiple imputation will be used. Additionally, sensitivity analyses based on complete-case datasets will be performed to assess the robustness and consistency of the findings.

## Results

This pilot study was funded by the *Bien vieillir chez soi au Bas-Saint-Laurent* action research program. Figure 1 illustrates the study timeline and the data collection process. Recruitment began in May 2024. We completed the data collection in February 2026. We began data cleaning and analysis in March 2026. As of September 2025, we enrolled 1 dyad of older adults and informal caregivers using MapIt, 5 dyads using Hygiene 2.0 or the combination of Hygiene 2.0 and MapIt, and 10 assistive technology advisors using at least one of both digital tools. The manuscript describing the results of the study is expected to be published at the beginning of 2027. manuscript

## Discussion

This PAR aims to cocreate the implementation of 2 innovative digital solutions, Hygiene 2.0 and MapIt, and to evaluate their impact on supporting older adults in the context of home care.

The i-PARIHS framework guided the development of the initial analytical questions (Multimedia Appendix 6) to describe mechanisms likely to influence the adoption of Hygiene 2.0 and MapIt. From an innovation perspective, the perceived utility, adaptability, and practical added value of these tools are expected to influence their acceptability and integration into everyday practice. Recipient characteristics, including users’ engagement, confidence, and support needs, are anticipated to shape how the tools are appropriated and used over time. The organizational and community context, such as available resources, service organization, and local priorities, may either enable or constrain implementation. Finally, facilitation processes are expected to play a central role in supporting user appropriation, aligning the tools with local practices and addressing emerging barriers. Together, these interacting dimensions help explain how adoption and implementation outcomes may vary across contexts.

In this pilot study, ethical and equity considerations guide all stages of implementation, shaping design decisions, deployment strategies, and ongoing reflexive monitoring. In this context, the use of free digital tools and the inclusion of a remote region enhance both financial and geographic accessibility. The project works to prevent ageism, digital exclusion, and paternalism by emphasizing informed consent, shared decision-making, and respect for older adults’ preferences. The approach adapts to diverse user needs—digital literacy, professional roles, and caregiving contexts—and ensures equitable access to training and support for the safe and meaningful adoption of MapIt and Hygiene 2.0. This approach aims to promote ethically responsible innovation and mitigate the risk of exacerbating existing digital and health disparities among older adults [42].

A key strength of this pilot study will be its implementation under uncontrolled real-world conditions, enabling the collection of both clinical and economic data reflective of actual practice. As emphasized by Brousselle et al [43], such effectiveness data are particularly valuable to users, clinicians, and decision-makers. While the small sample size (n=66) may limit generalizability, it provides a foundation for building a larger evidence base. The early insights generated by this research can inform future, larger-scale evaluations of Hygiene 2.0 and MapIt. This pilot study is designed to yield rapidly applicable data for various stakeholders involved in the home adaptation process, health care professionals, decision-makers, informal caregivers, and older adults themselves. These findings can directly inform shared decision-making, particularly among occupational therapists, older adults, and policy actors, by illustrating how innovations affect quality of life and satisfaction with aging in place.

Considering an aging population, ensuring that older adults can remain at home safely has become a public health priority. However, this cannot be achieved without engaging those directly affected. The PAR approach [44] is particularly relevant here, as it empowers older adults by actively involving them in the research process [45]. This collaborative model aligns the research process with the lived experiences, needs, and knowledge of both older adults and assistive technology advisors, producing more nuanced, context-sensitive data.

By coconstructing the implementation of these innovations, the pilot study aims to enhance both support and intervention quality in home adaptations. Moreover, PAR fosters the agency of older adults, giving them an active role in shaping the tools and processes designed for them [46]. This user involvement not only supports the adoption, scalability, and sustainability of innovations, such as Hygiene 2.0 and MapIt, but also ensures that they are adaptable to local contexts and responsive to evolving needs. Furthermore, engaging older adults directly can improve the acceptability of innovations [47], as their experiential knowledge often eludes traditional research methods. Thus, PAR provides a means to capture and integrate this knowledge into the design of more relevant and effective solutions [44].

Despite the study’s strengths, certain limitations, such as methodological and contextual challenges, must be acknowledged. Recruitment may prove more challenging than anticipated, as the projected sample size is based on untested data collection procedures, and actual participation rates may differ significantly. Recruitment and engagement of older adults may be influenced by variability in digital literacy and access to technological and internet resources, particularly in rural settings. To mitigate these barriers, all equipment required for the use of MapIt and Hygiene 2.0 is loaned free of charge by Université TÉLUQ. Although the study aims to promote the independent use of Hygiene 2.0 by older adults, it is recognized that some individuals may require support. Therefore, family caregivers and advisors from community organizations are also included as target users to assist older adults when needed, without undermining their autonomy. Additional socioeconomic and organizational challenges are addressed through flexible participation modalities, integration of the tools within existing care and community practices, and reliance on established institutional partnerships to reduce professional burden and support feasibility. Consequently, findings regarding the impact of the innovations should be interpreted with caution. In the event of low recruitment among older adults, qualitative interviews with assistive technology advisors will provide essential context for understanding barriers to adoption and implementation.

The use of a convenience sample entails potential risks of selection, as individuals who are more available or motivated are more likely to participate. This situation may compromise the representativeness of the sample and, consequently, the validity of the findings. To mitigate these limitations, several strategies will be implemented: (1) thoroughly documenting sample characteristics and (2) employing triangulation of data sources and collection methods within the convergent mixed methods design. These measures aim to strengthen the credibility of the results and enable a rigorous assessment of the transferability of the findings to other contexts.

The successful implementation of this project will provide a unique experience in cocreating and deploying 2 digital innovations in regions remote from urban centers. Implementing Hygiene 2.0 and MapIt offers significant added value. Hygiene 2.0 supports early prevention by identifying appropriate equipment or the need for occupational therapy consultation at the earliest stage. MapIt enables remote measurements, reduces unnecessary home visits, and facilitates measurements in hard-to-access areas, while providing visual support for space reorganization. These features translate into clinical and economic efficiency gains, including improved prevention, enhanced service performance, cost savings for the health care system, and potential relief of the financial burden on older adults and their caregivers.

### Conclusion

In conclusion, this pilot PAR provides an opportunity to cocreate and evaluate the implementation of Hygiene 2.0 and MapIt while fostering a more inclusive and collaborative approach to innovation. By involving older adults and other stakeholders directly in the research and implementation processes, this pilot study will contribute to the development and implementation of digital innovations that are better aligned with the realities of aging in place. Beyond the immediate context, the findings will offer actionable insights into the conditions, facilitators, and challenges associated with integrating digital tools into home adaptation practices. These insights may inform future provincial and national strategies for home adaptation and digital health by supporting evidence-informed decisions regarding the adoption of user-centered technologies within publicly funded programs. Knowledge transfer activities generated through this project will extend beyond the regional scope through dissemination within professional and policy networks, targeted training initiatives, and dialogue with decision-makers, thereby supporting scalability and sustainability. Ultimately, this work aims to strengthen health systems’ capacity to respond to demographic aging, reduce inequities in access to rehabilitation services, and foster a sustained culture of innovation in home adaptation.

## Supplementary material

10.2196/84680 Multimedia Appendix 1 Hygiene 2.0 posters.

10.2196/84680 Multimedia Appendix 2 Training modules.

10.2196/84680 Multimedia Appendix 3 Interview guide.

10.2196/84680 Multimedia Appendix 4 Logbook and usage grid.

10.2196/84680 Multimedia Appendix 5 Appraisal questionnaire.

10.2196/84680 Multimedia Appendix 6 Initial analytical questions.

## Abbreviations

BSL: Bas-Saint-Laurent
CHEERS: Consolidated Health Economic Evaluation Reporting Standards
CoPaQ: Costs for Patients Questionnaire
HAP: Home Adaptation Program
i-PARIHS: integrated-Promoting Action on Research Implementation in Health Services
ICC: intraclass correlation coefficient
PAR: participatory research action
QALY: quality-adjusted life year
QUEST: Quebec User Evaluation of Satisfaction with Assistive Technology
SF-6Dv2: Short Form-6 Dimensions, version 2
SUS: System Usability Scale
ZBI: Zarit Burden Interview
