# Supplementary material for: Cocreation, Implementation, and Evaluation of Two Digital Innovations to Improve Home Adaptation for Older Adults: Protocol for an Action Research Pilot Study
Source: JMIR Res Protoc. 2026 Sep 3;15:e84680. doi: 10.2196/84680 (PMC13540621; doi:10.2196/84680)
Supplement: Supplementary file 1 [file resprot-v15-e84680-s001.pdf]

DES DIFFICULTÉS À SE LAVER?

PEUR DE TOMBER?

DES QUESTIONS SUR LES BANCS DE BAIN?

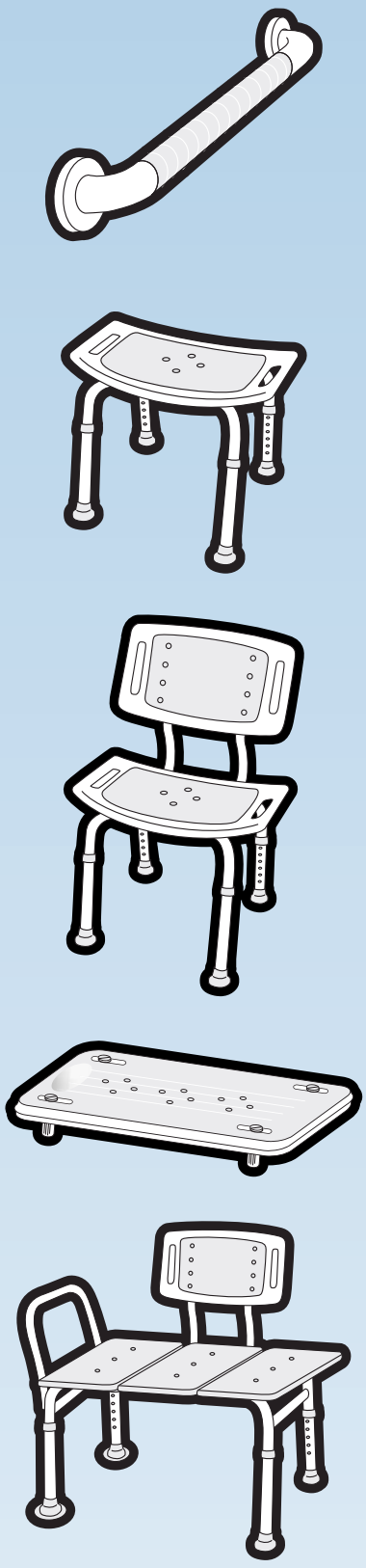

RÉPONDEZ À DES QUESTIONS SIMPLES

sur vos habitudes, vos capacités et votre salle de bain.

OBTENEZ DES RECOMMENDATIONS CLAIRES

sur des aménagements, des équipements ou des service en ergothérapie.

Suivez le lien web

h20.manonguay.ca

Ou scannez le code QR

H2O

Manon GUAY, erg., Ph. D.
manonguay.ca

CC BY NC ND **2024**

Bien vieillir chez soi
RECHERCHE-ACTION

Comment utiliser les **BARRES D'APPUI**

DÉMONSTRATION VIDÉO

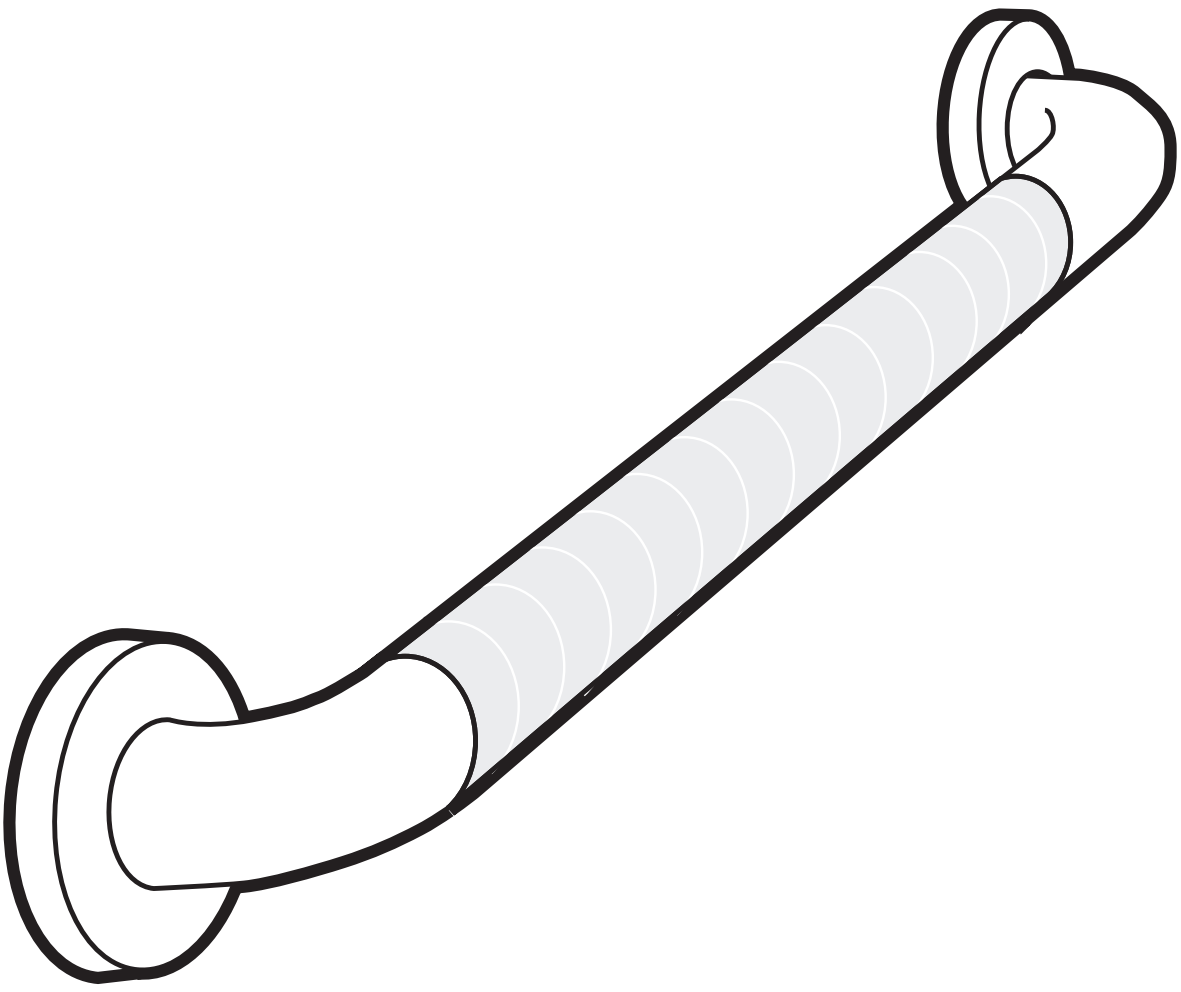

Suivez le lien web

h20.manonguay.ca/
videos/**barre**

Ou scannez le code QR

Hygiene 2.0

Bien vieillir chez soi
RECHERCHE-ACTION

Manon GUAY, erg., Ph. D.
manonguay.ca

CC BY NC ND 2024

Comment utiliser le

TABOURET

DÉMONSTRATION VIDÉO

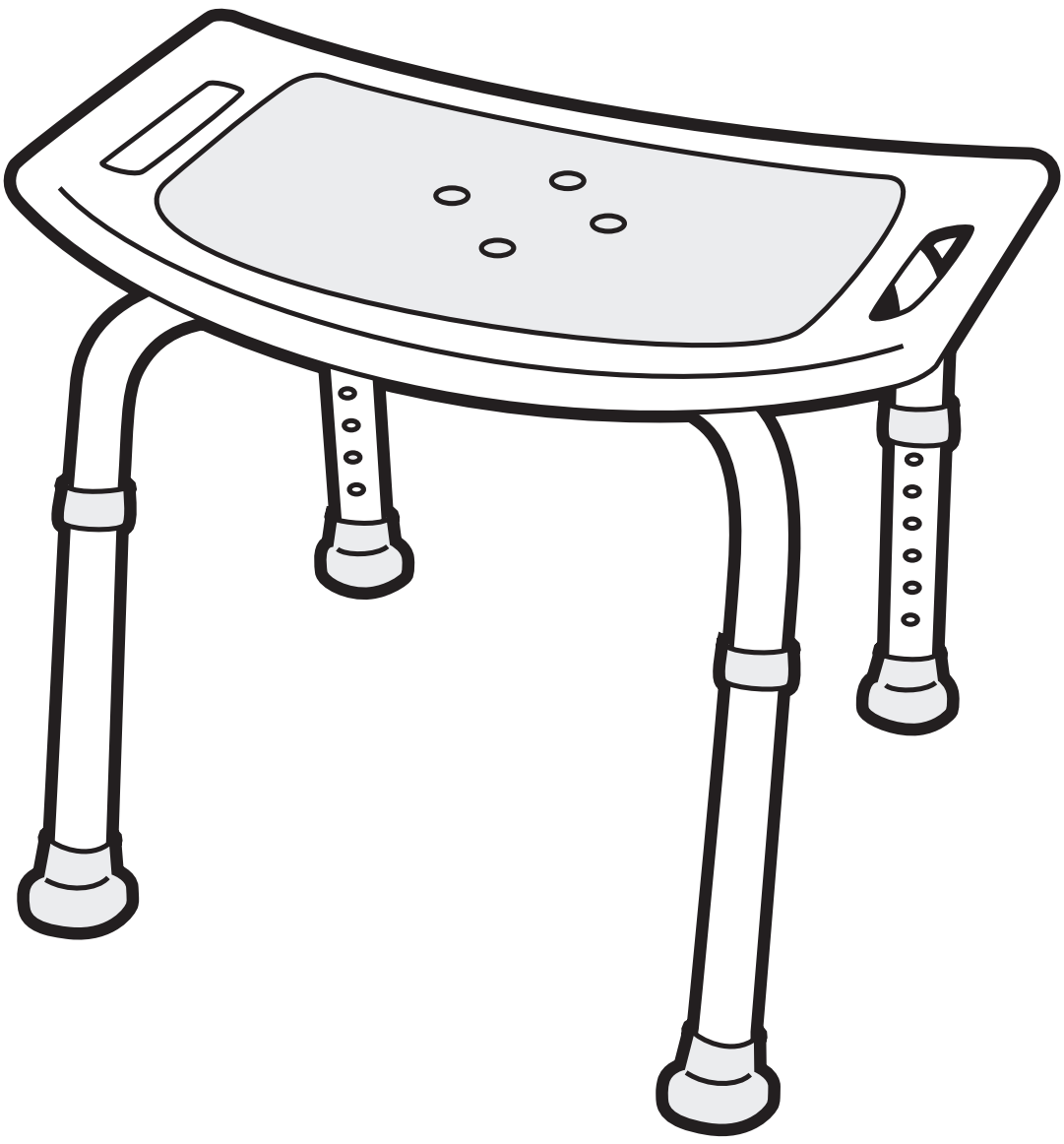

Suivez le lien web

h20.manonguay.ca/
videos/**tabouret**

Ou scannez le code QR

Hygiene 2.0

Bien vieillir chez soi
RECHERCHE-ACTION

Manon GUAY, erg., Ph. D.
manonguay.ca

2024

Comment utiliser le

SIÈGE AVEC DOSSIER

DÉMONSTRATION VIDÉO

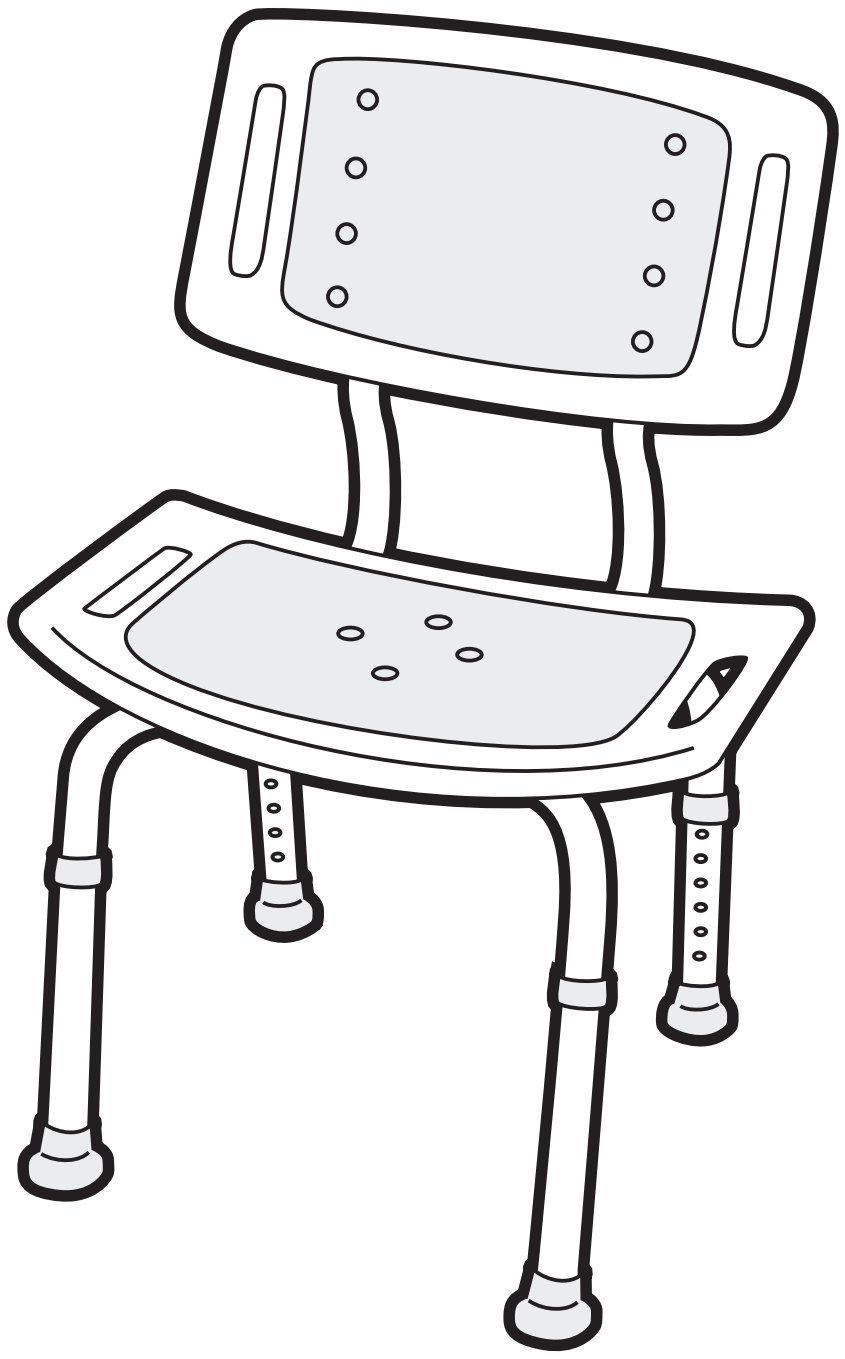

Suivez le lien web

h20.manonguay.ca/
videos/**siege**

Ou scannez le code QR

Hygiène 2.0

Bien vieillir chez soi
RECHERCHE-ACTION

Manon GUAY, erg., Ph. D.
manonguay.ca

2024

Comment utiliser la

PLANCHE DE TRANSFERT

DÉMONSTRATION VIDÉO

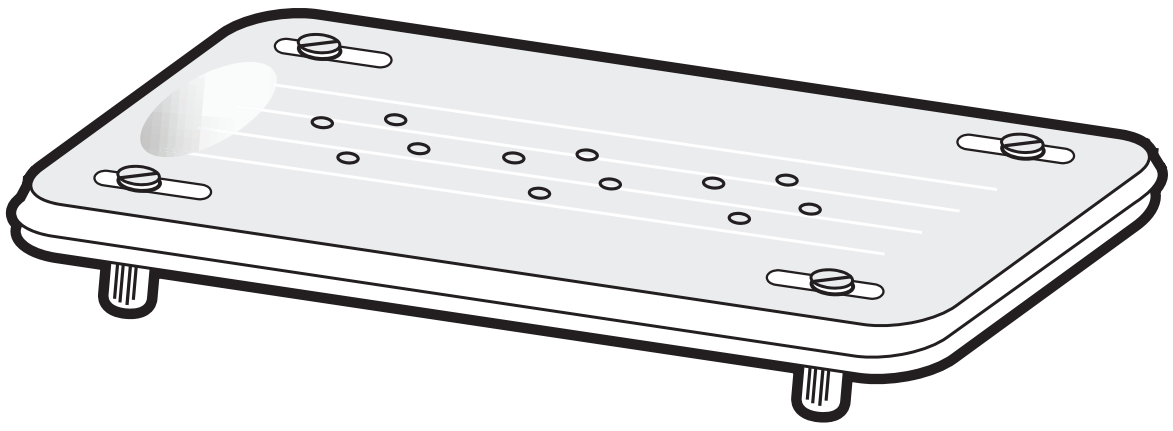

Suivez le lien web

h20.manonguay.ca/
videos/**planche**

Ou scannez le code QR

Hygiene 2.0

Bien vieillir chez soi
RECHERCHE-ACTION

Manon GUAY, erg., Ph. D.
manonguay.ca

CC BY NC ND 2024

Comment utiliser le

FAUTEUIL DE TRANSFERT

DÉMONSTRATION VIDÉO

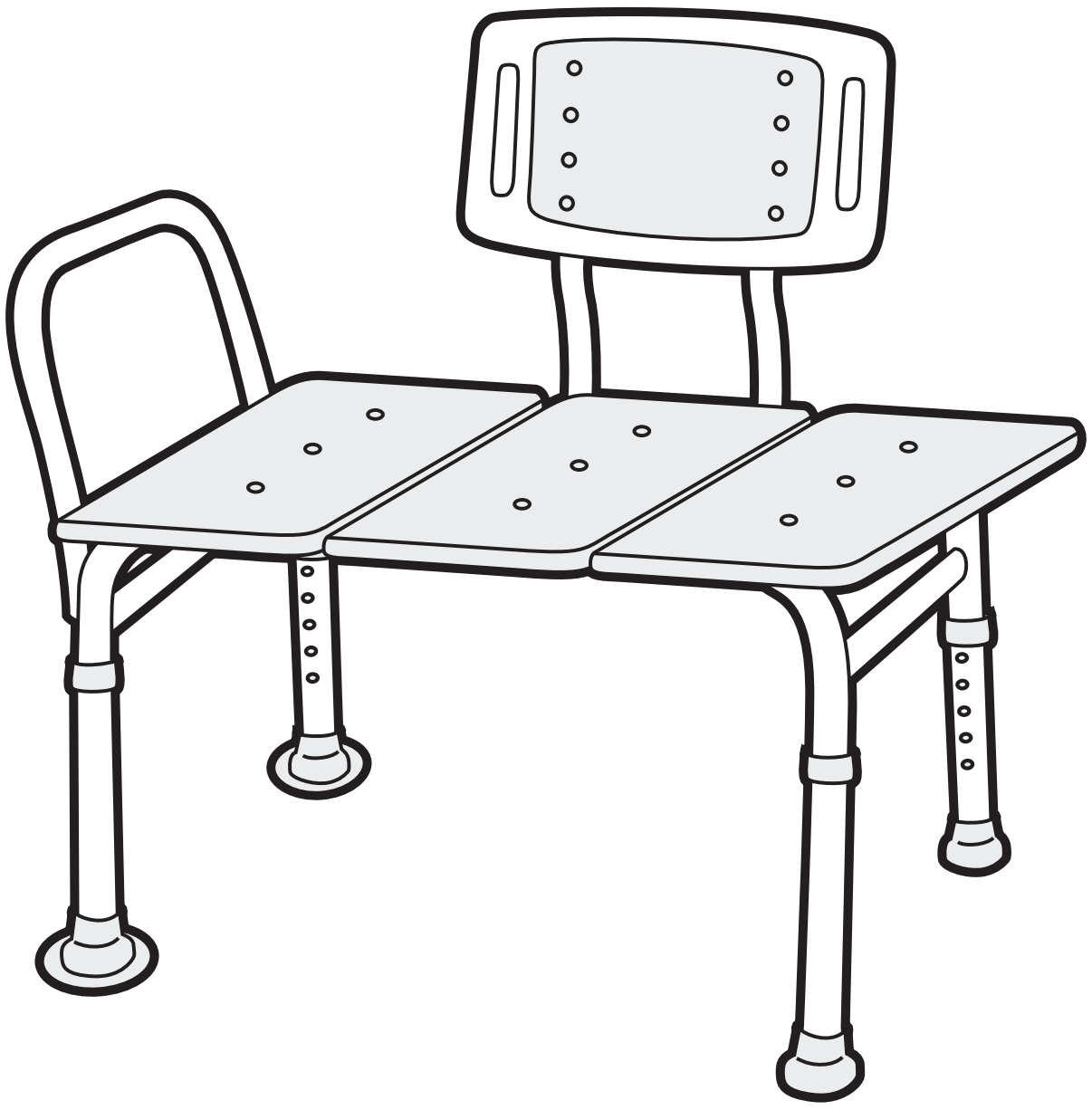

Suivez le lien web

h20.manonguay.ca/ videos/**fauteuil**

Ou scannez le code QR

Hygiene 2.0

Bien vieillir chez soi

RECHERCHE-ACTION

Manon GUAY, erg., Ph. D.
manonguay.ca

2024
