# Supplementary material for: Cocreation, Implementation, and Evaluation of Two Digital Innovations to Improve Home Adaptation for Older Adults: Protocol for an Action Research Pilot Study
Source: JMIR Res Protoc. 2026 Sep 3;15:e84680. doi: 10.2196/84680 (PMC13540621; doi:10.2196/84680)
Supplement: Supplementary file 2 [file resprot-v15-e84680-s002.pdf]

TRAINING MODULE

This three-hour training is free, entirely online, and self-directed. MapIt is used to create a 3D model of a person's built environment. The training consists of five compulsory modules and one optional module:

Main Modules

1. MapIt and How it's Used

2. Scan With the MapIt Mobile App

3. Transfer the 3D Models to a Computer or Partner

4. When and Why to Use MapIt

5. Automatic or Customized Measurement

Optional Module:

6. Practise Using MapIt (complementary activity)

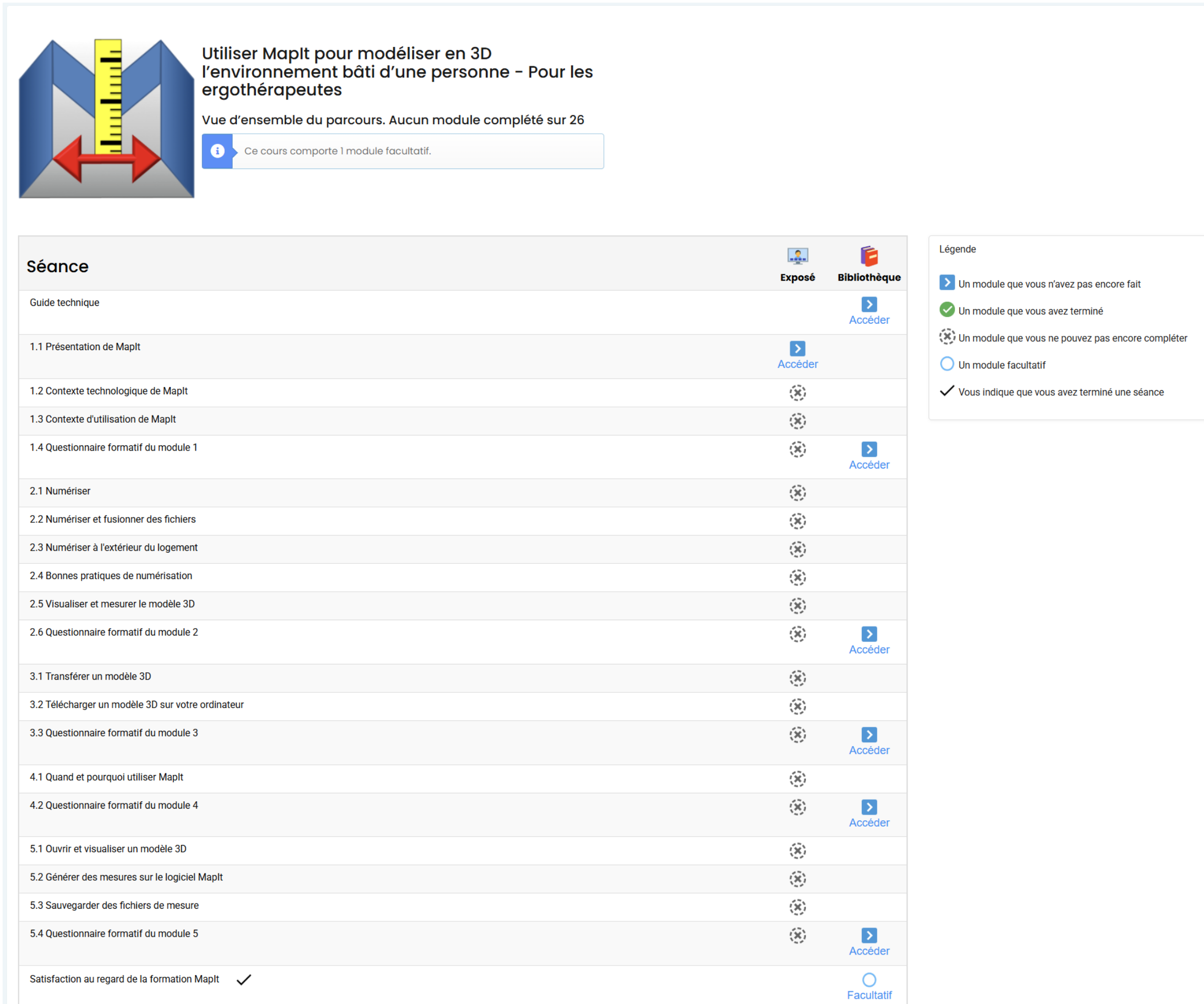

Utiliser MapIt pour modéliser en 3D l'environnement bâti d'une personne – Pour les ergothérapeutes

Vue d'ensemble du parcours. Aucun module complété sur 26

Ce cours comporte 1 module facultatif.

Séance	Exposé	Bibliothèque
Guide technique		Accéder
1.1 Présentation de MapIt	Accéder	
1.2 Contexte technologique de MapIt		
1.3 Contexte d'utilisation de MapIt		
1.4 Questionnaire formatif du module 1		Accéder
2.1 Numériser		
2.2 Numériser et fusionner des fichiers		
2.3 Numériser à l'extérieur du logement		
2.4 Bonnes pratiques de numérisation		
2.5 Visualiser et mesurer le modèle 3D		
2.6 Questionnaire formatif du module 2		Accéder
3.1 Transférer un modèle 3D		
3.2 Télécharger un modèle 3D sur votre ordinateur		
3.3 Questionnaire formatif du module 3		Accéder
4.1 Quand et pourquoi utiliser MapIt		
4.2 Questionnaire formatif du module 4		Accéder
5.1 Ouvrir et visualiser un modèle 3D		
5.2 Générer des mesures sur le logiciel MapIt		
5.3 Sauvegarder des fichiers de mesure		
5.4 Questionnaire formatif du module 5		Accéder
Satisfaction au regard de la formation MapIt ✓		Facultatif

Légende

- Un module que vous n'avez pas encore fait
- Un module que vous avez terminé
- Un module que vous ne pouvez pas encore compléter
- Un module facultatif
- Vous indique que vous avez terminé une séance

Screenshot of the training on the website of the Order of Occupational Therapists of Quebec
