# Supplementary material for: Cocreation, Implementation, and Evaluation of Two Digital Innovations to Improve Home Adaptation for Older Adults: Protocol for an Action Research Pilot Study
Source: JMIR Res Protoc. 2026 Sep 3;15:e84680. doi: 10.2196/84680 (PMC13540621; doi:10.2196/84680)
Supplement: Supplementary file 3 [file resprot-v15-e84680-s003.pdf]

INTERVIEW GUIDE
[Notes for the interviewer]

Hello, thank you for taking the time to speak with me today. The objective of this project is to co-create the implementation and evaluate the effects of the Hygiene 2.0 and MapIt innovations in supporting seniors who have challenges with bathing or showering or who are engaged in a process of adapting their home in the Bas-Saint-Laurent (BSL), as well as the stakeholders involved.

This interview aims to explore your experience using (MapIt or Hygiene 2.0) or adapting your home. I'm interested in hearing about your use of the innovation and how it has affected the adaptation of your home. The interview lasts between 15 and 45 minutes and is recorded in audio format. Only the project's researchers will have access to the recording.

[*Intent and consent form reminders*]

Of course, everything you tell me today will remain confidential, meaning that no personal data that could identify you, such as your name, age, or place of residence, will be disseminated. There are no right or wrong answers, we are interested in your point of view. You can withdraw from the interview at any time.

First Interview [Senior, Dyad], All Interviews [Advisor]

1. **[Senior or Dyad]** What problems did you encounter that led to the adaptation of your bathroom or your home?

2. **[Advisor]** Tell me about the context of your interventions.

3. [*If the person has used an innovation. If not, go to Question 5*] Tell me about [MapIt or Hygiene 2.0] and the context in which you used it.

   Sub-question track for the interview, if necessary:

   a. *How did it go?*

   b. *What device did you use?*

   c. *How long did it take to use the innovation?*

   d. *Was the innovation useful?*

   e. *[For H2.0] What do you think of H2.0's recommendation?*

   f. *[For MapIt] Do you think that using MapIt has saved you any time in terms of your home adaptations? (YES or NO)*

   - *How and why? (Have you avoided any visits or calls, wasted time on technical problems, has the MapIt map been used outside of visits, etc.)*

- *Estimated time saved or lost (specify call duration, visit duration, e.g., a 1-hour visit, 10-minute call)*
- *[If yes] Estimated number of visits avoided*
- *[If yes] Estimated number of calls avoided*

4. Describe your level of comfort in using the innovation.
   a. *Have you encountered any problems while using the innovation?*

[If the adaptation has already been completed or is in progress]

5. What did you find more difficult in the adaptation/recommendation process?
6. What made the adaptation/recommendation process easier?
7. *[If control group, skip this question]* What do you think the impact of using [Hygiene 2.0 or MapIt] has been? [*May be relevant even if the person did not directly use the innovation, for example if a consultant used it for them*]
8. **[Senior or Dyad]** What costs did you incur in adapting your bathroom/home?
9. **[Senior or Dyad]** Can you describe the impact on your life of adapting your bathroom/home?
10. **[Senior or Dyad]** Have you ever fallen in your home and if so, can you tell me about it?
11. Are there any other elements you wish to address?

Subsequent Interviews—Seniors and Dyad

1. How does the accommodation/recommendation process work?

    a. What did you find more difficult in the adaptation/recommendation process?

    b. What made the adaptation/recommendation process easier?

2. *[If control group, skip this question]* What do you think the impact of using [Hygiene 2.0 or MapIt] has been? [*May be relevant even if the person did not directly use the innovation, for example if a consultant used it for them*]

3. What costs did you incur in adapting your bathroom/home? [*What purchases? How much?]*

4. Can you describe the impact on your life of adapting your bathroom/home?

5. Have you fallen one or more times in your home since the last meeting and, if so, can you tell me about it? [*Where? With or without technical aids or adaptations?]*

6. Are there any other elements you wish to address?
