# Supplementary material for: Cocreation, Implementation, and Evaluation of Two Digital Innovations to Improve Home Adaptation for Older Adults: Protocol for an Action Research Pilot Study
Source: JMIR Res Protoc. 2026 Sep 3;15:e84680. doi: 10.2196/84680 (PMC13540621; doi:10.2196/84680)
Supplement: Supplementary file 4 [file resprot-v15-e84680-s004.pdf]

Participant: ________

GRID DOCUMENTING THE USE OF MAPIT

<table>
<tr><td rowspan="10">Click or tap here to enter a date.</td><td>Distance from the user's residence</td><td colspan="2">km (between the user's residence and the occupational therapist's workplace)</td></tr>
<tr><td>Age of the user</td><td colspan="2">Years</td></tr>
<tr><td></td><td colspan="2"></td></tr>
<tr><td>Type(s) of users</td><td>☐ Physical impairment – adult
☐ Physical impairment – senior
☐ Age-related disorder (e.g., frailty, neurodegenerative disease)</td><td>☐ Autism spectrum disorder
☐ Intellectual impairment
☐ Visual and hearing impairment
☐ Palliative care
☐ Other:____________</td></tr>
<tr><td>Check what you used in this case</td><td colspan="2">☐ Mobile application ☐ Desktop software</td></tr>
<tr><td>+ Aspects/Strong points</td><td colspan="2"></td></tr>
<tr><td>– Aspects/Weaknesses</td><td colspan="2"></td></tr>
<tr><td>MapIt enabled you to save a trip</td><td colspan="2">☐ Yes ☐ No</td></tr>
<tr><td>MapIt saved you time</td><td colspan="2">☐ Yes ☐ No</td></tr>
<tr><td>If so, how much time (approximately) and why</td><td colspan="2"></td></tr>
</table>
