# Supplementary material for: Cocreation, Implementation, and Evaluation of Two Digital Innovations to Improve Home Adaptation for Older Adults: Protocol for an Action Research Pilot Study
Source: JMIR Res Protoc. 2026 Sep 3;15:e84680. doi: 10.2196/84680 (PMC13540621; doi:10.2196/84680)
Supplement: Supplementary file 5 [file resprot-v15-e84680-s005.pdf]

LimeSurvey

This questionnaire, which takes about 10 minutes to complete, will allow the research team that developed Hygiene 2.0 to assess whether it is useful and simple.

Your responses will remain anonymous.

Part A: Navigation Number

A1. **If you still have access to it, enter the seven alphanumeric characters at the bottom right of the Hygiene 2.0 screen.**

Note: No matter the page, these characters are always the same for the same navigation

Part B: Hygiene 2.0 Usability

B1. **Think back to your navigation in Hygiene 2.0, please indicate your level of agreement on a scale of 1 to 5.**

(From 1- Strongly disagree to 5 - Strongly agree.)

	Strongly disagree	1. Slightly disagree	3Neutral	4. Slight ly agree	Strongly agree
1. I would like to use Hygiene 2.0 frequently	☐	☐	☐	☐	☐
2. Hygiene 2.0 is unnecessarily complex	☐	☐	☐	☐	☐
3. Hygiene 2.0 is easy to use	☐	☐	☐	☐	☐
4. I would need the support of a technician to be able to use Hygiene 2.0	☐	☐	☐	☐	☐
5. The various features of Hygiene 2.0 are well integrated	☐	☐	☐	☐	☐
6. There are too many inconsistencies in Hygiene 2.0	☐	☐	☐	☐	☐
7. Most people will learn to use Hygiene 2.0 very quickly	☐	☐	☐	☐	☐
8. Hygiene 2.0 is very cumbersome to use	☐	☐	☐	☐	☐
9. I felt very confident using Hygiene 2.0	☐	☐	☐	☐	☐
10. I had to learn a lot before I could use Hygiene 2.0	☐	☐	☐	☐	☐

Part C: Assessment of the H2.0 Application

C1. **Was Hygiene 2.0 useful to you?**

Yes ☐

No ☐

C2. **Comment:**

C3. **Would you recommend Hygiene 2.0?**

- Yes ☐
- No ☐

C4. **Comment:**

Part D: Obtaining the Hygiene 2.0 Link

D1. **How did you hear about Hygiene 2.0?**

Add a comment if you wish to do so. If you answer "Other", please explain.

- I received a QR code or web link ☐
- I found the web link by myself ☐
- Other (please explain) ☐

D2. **Who gave you the QR code or the web link to Hygiene 2.0?**

Add a comment if you wish to do so. If you answer "Other", please explain.

A person at the CLSC equipment loan desk

Comment

A person who visited me at home (Occupational Therapist, Physiotherapist, Nurse, etc.)

Comment

A person at the pharmacy

Comment

Someone in my doctor’s office or my specialized nurse practitioner

Comment

Other (please explain)

Comment

D3. Where did you find the QR code or the web link to Hygiene 2.0?

Add a comment if you wish to do so. If you answer “Other”, please explain.

At the doctor’s office

Comment

At the CLSC

Comment

At the pharmacy

Comment

At the post office

Comment

LimeSurvey

At the health and solidarity cooperative

Comment

In a social economy business (SEB)

Comment

At the Volunteer Action Centre (VAC)

Comment

In a newspaper

Comment

In a seniors' residence

Comment

On the Internet

Comment

Other (please explain)

Comment

LimeSurvey

Part E: Region of Residence

E1. What region do you live in?

Region of your principal residence

- Abitibi-Témiscamingue ☐
- Bas-Saint-Laurent ☐
- Capitale-Nationale ☐
- Centre-du-Québec ☐
- Chaudière-Appalaches ☐
- Côte-Nord ☐
- Eastern Townships ☐
- Gaspésie–Îles-de-la-Madeleine ☐
- Lanaudière ☐
- Laurentians ☐
- Laval ☐
- Mauricie ☐
- Montérégie ☐
- Montreal ☐
- Northern Quebec ☐
- Ottawa ☐
- Saguenay–Lac-Saint-Jean ☐

E2. **Specifically, in which Bas-Saint-Laurent administrative region do you live?**

Choose the administrative region of your principal residence

- Kamouraska ☐
- Rivière-du-Loup ☐
- Témiscouata ☐
- Basques ☐
- Rimouski ☐
- Matane (Matanie) ☐
- Mitis ☐
- Matapédia ☐

Part F: Consent to be Contacted

F1. **I consent to be contacted by Professor Karine Latulippe, occupational therapist, or a member of her team at Université TÉLUQ, to participate in a research project.**

- Yes ☐
- No ☐

F2. **Please complete the following information:**

Last name:

First name:

Contact phone number:

Contact email:

Thank you so much for completing this questionnaire!

Your participation helps to advance research.
