# Supplementary material for: Cocreation, Implementation, and Evaluation of Two Digital Innovations to Improve Home Adaptation for Older Adults: Protocol for an Action Research Pilot Study
Source: JMIR Res Protoc. 2026 Sep 3;15:e84680. doi: 10.2196/84680 (PMC13540621; doi:10.2196/84680)
Supplement: Supplementary file 6 [file resprot-v15-e84680-s006.docx]

**Initial analytical questions**

Implementation of innovations

Model i-PARIHS* 🡪 SI = Fac^n^(I+R+C)

Where

SI = Successful implementation (Implantation réussie)

Facn = Facilitation

I = Innovation

R = Recipients

C = Context

1. What was the starting point for the *innovations* to be implemented? (See question 11 for the assessment of evolution)
2. Who were the *recipients* of the innovations?
3. What are the *contexts* in which the innovations have been used?
   a. More specifically, what are the contexts of intervention (type of clientele, environment, type of adaptation, etc.)?
4. What are the *contexts* in which innovations have not been used (or useful) in the end?
   a. More specifically, what are the contexts of intervention (type of clientele, environment, type of adaptation, etc.)?
5. What were the steps taken to facilitate the implementation of innovations?
   a. What were the barriers to the implementation of innovations?
   b. Who were the facilitators in the implementation of the innovations?

Effects of innovations

6. What are the effects of innovations on seniors?
7. What are the effects of innovations on caregivers?
8. What are the effects of innovations on stakeholders in the cross-sectoral ecosystem?
9. Have innovations influenced the time of interventions (increased, decreased, no influence)?

Usability of innovations

10. What is the usability of innovations according to the people who have used them?
    a. Have users been able to get results?
    b. What do users think of these results?
    c. What do users think about the time required to use innovations?
    d. What were the mistakes made and their consequences?
    e. What is the overall satisfaction of users with innovations?
11. How have innovations evolved over the course of implementation?

Bathroom/home adaptation process

12. How did the home adaptations go?
    a. What were the barriers to home adaptation?
    b. Who were the facilitators of home adaptation?
13. What are the costs involved in adapting the home?
14. What are the effects of home adaptations on the lives of seniors and their loved ones?
